# A systematic review of economic evaluations of pharmacological treatments for adults with chronic migraine

**DOI:** 10.1186/s10194-022-01492-y

**Published:** 2022-09-16

**Authors:** Saval Khanal, Martin Underwood, Seyran Naghdi, Anna Brown, Callum Duncan, Manjit Matharu, Hema Mistry

**Affiliations:** 1 Warwick Evidence, Warwick Medical School, University of Warwick, Coventry, CV4 7AL UK; 2 Warwick Clinical Trials Unit, Warwick Medical School, University of Warwick, Coventry, CV4 7AL UK; 3 University Hospitals Coventry and Warwickshire NHS Trust, Coventry, CV2 2DX UK; 4 Department of Neurology, NHS Grampian, Aberdeen Royal Infirmary, Aberdeen, AB25 2ZN UK; 5 National Hospital for Neurology and Neurosurgery, University College London Hospitals NHS Trust, London, WC1N 3BG UK

**Keywords:** Headache, Migraine, Migraine prevention, Botox, CGRP monoclonal antibodies, Erenumab, Galcanezumab, Fremanezumab, Cost-effectiveness

## Abstract

**Background and aims:**

Chronic migraine is a common neurovascular brain disorder with substantial economic costs. We performed a systematic review to identify economic evaluations of pharmacological treatments for adults with chronic migraine.

**Methods:**

We undertook systematic literature searches using terms for migraine/headache and prophylactic drug interventions, combined with economic/cost terms where appropriate. Using inclusion and exclusion criteria, two reviewers independently assessed the citations and abstracts, and full-text articles were retrieved. A review of study characteristics and methodological quality was assessed.

**Results:**

Sixteen citations met the inclusion criteria and were model-based cost-utility studies evaluating: Botox (*n* = 6); Erenumab (*n* = 8); Fremanezumab (*n* = 2); and Galcanezumab (*n* = 1) as the main treatment. They varied in their use of comparators, perspective, and model type. Botox was cost-effective compared to placebo with an incremental cost-effectiveness ratio (ICER) ranging between £15,028 (€17,720) and £16,598 (€19,572). Erenumab, Fremanezumab and Galcanezumab when compared to Botox, was associated with ICERs ranging between £59,712 ($81,080) and £182,128 (€218,870), with the ICERs above the most common willingness-to-pay thresholds (WTPs). But they were cost-effective within the commonly used WTPs among the population for whom the previous treatments including Botox were failed. Three studies compared the cost-effectiveness of Erenumab against the placebo and found that Erenumab was dominant. All studies performed sensitivity analyses to check the robustness of their results. None of the findings from the included articles were generalisable and none of the included studies fulfilled all the criteria mentioned in the CHEERS 2022 reporting checklist and Phillips’s checklist for economic models.

**Conclusions:**

Evidence to support the cost-effectiveness of pharmacological treatments of chronic migraine in the adult population using Botox and Erenumab were identified. Our findings suggest that both Botox and Erenumab, are cost-effective compared to placebo; although Erenumab had more incremental economic benefits compared to Botox, the ICERs were above the most common willingness-to-pay thresholds. Hence, Erenumab might be an acceptable treatment for chronic migraine for patients whom other treatments such as Botox do not work. Further research is needed to help characterise the data to adequately structure and parameterise an economic model to support decision-making for chronic migraine therapies.

**Supplementary Information:**

The online version contains supplementary material available at 10.1186/s10194-022-01492-y.

## Introduction

Migraine is the world’s second most common disabling disorder [1] and the top cause of years lived with disability in those aged 15-49 years [2]. Chronic migraine is a debilitating neurological condition. It is one of the most important non-transmissible disorders. The overall global prevalence of chronic migraine is between 1.4 -2.2% [1]. Migraine has huge social and economic impact on the society. For example, in the United Kingdom (UK), one out of each six adults are affected by migraine. Most commonly these are young adults with work and family commitments [3], and the economic burden in the UK is over £1.5 billion per year [3]. The definition of chronic migraine has developed over time. Version three (2018) of the international classification of headache disorder defines chronic migraine as *‘Headache occurring on 15 or more days/month for more than three months, which, on at least eight days/month, has the features of migraine headache’* [4]*.* For those affected and for the healthcare system(s), chronic migraine is a financially burdensome condition [5–7], this includes both direct costs such as hospitalisation and medications, and indirect costs resulting from work presenteeism and absenteeism. Prophylactic drug treatments are a core part of the management of chronic migraine [2].

The health technology assessment organisations and other decision-making bodies may use the evidence on economic evaluations to learn the economic value of the available treatments. Such kind of review should comprehensively capture the information about the cost and benefits associated with those treatments. Our literature search identified two such reviews about migraine treatment. In the first one, Yu et al. (2009) in a review of economic evaluations of drug treatments for migraine, identified three cost-effectiveness studies of drugs for migraine prophylaxis [8]. All three were studies of the episodic migraine [9–11]. More recently (2020), a second review about the cost-effectiveness of the treatment for migraine including chronic and episodic migraines was published, but the inclusion criteria were limited to articles published from the UK and the Ireland [12]. With the advent of newer drugs, such as calcitonin gene-related peptide monoclonal antibodies (CGRP MAbs), and increased use of botulinum toxin type A (BTA) or OnabotulinumtoxinA or Botox, all of which are principally targeted at people with chronic migraine there is a need for an updated review to identify what is known about the cost-effectiveness of prophylactic drugs when used for chronic migraine at a global level. Hence, we have conducted a systematic review to identify all economic evaluations of prophylactic drug treatments for the treatment of chronic migraine, regardless of study design (trial-based economic evaluation or model-based economic evaluation).

## Methods

### Search strategy

Working with an information specialist (AB), we developed a search initially constructed in MEDLINE, using both free text keywords and thesaurus (MeSH) terms for migraine/headache and prophylactic drug interventions (including named drugs of interest). These were combined with a search filter for economic and cost studies. No language nor date limits were applied. Search strategies in economics/HTA specific sources included only terms for migraine/headache, with the addition of general terms for drug treatment or prevention in some cases. An example of the MEDLINE search strategy can be found in Additional file 1: Appendix 1. This search strategy was checked by another information specialist, not otherwise involved in the project, for errors in spelling, search syntax or structure prior to being run in MEDLINE and translated to other databases. The following bibliographic databases were searched on 6th September 2021.

- MEDLINE All, 1946 to September 03, 2021 (via Ovid)
- Embase Classic + Embase, 1947 to 2021 September 03 (via Ovid)
- EconLit (via EbscoHost)
- NHS Economic Evaluation Database (NHS EED) (via CRD website)
- Health Technology Assessment (HTA) database (via CRD website)
- International HTA database (via INAHTA website)
- Cost-effectiveness Analysis Registry (via Tufts Medical Center website)
- EconPapers (via Research Papers in Economics (RePEc))

These were supplemented by targeted internet searches using Google and Google Scholar, on 13th September 2021. The websites of the National Institute for Health and Care Excellence (NICE), Scottish Medicines Consortium (SMC), All Wales Medicine Strategy Group (AWMSG) and Canadian Agency for Drugs and Technology in Health (CADTH) were also searched on 7th September 2021 for publications relating to migraine or headache. We summarised the published journal articles separately from the reports, as the latter will not have had a formal peer-reviewed process. We used EndNote X20 to manage references including the removal of duplicates.

The review and updates were performed in accordance with the methodological principles of conduct for systematic reviews as reported in the Preferred Reporting Items for Systematic Reviews and Meta-Analyses (PRISMA) checklist [13]. No ethical approval was required. The protocol is registered with the international prospective register of systematic reviews (PROSPERO) database (reference number: CRD42021265995).

We included full economic evaluations in which both the costs and the outcomes of interventions and alternatives are examined including both trial-based and model-based evaluations. We included four types of economic evaluations: cost-benefit analysis [CBA], cost-consequence analysis [CCA], cost-effectiveness analysis [CEA], and cost-utility analysis [CUA]. We excluded partial economic evaluations, systematic reviews, meta-analyses, qualitative studies, conference abstracts, editorials, short commentary and study protocols.

#### Population

Adults with chronic migraine, where the headache occurred for 15 or more days/month for more than 3 months.

#### Interventions

Pharmacotherapy that is used as prophylactic drugs to treat chronic migraine such as CGRP Mabs, BTA, anti-depressants, angiotensin converting enzyme (ACE) inhibitors, angiotensin receptor blockers, beta-blockers, calcium channel blockers, pizotifen, and anticonvulsants (topiramate, valproate/divalproex, gabapentin).

#### Comparators

Placebo, usual care, or other prophylactic drugs. Any articles comparing pharmacotherapy with non-pharmacological interventions were excluded.

#### Outcomes

Measures included but not limited to: headache/migraine days, headache-related quality of life including Headache Impact Test 6 (HIT-6), Migraine Disability Assessment Score (MIDAS), and migraine specific quality of life, acute treatment use, headache intensity and duration, health service activity, days lost from usual activities, incremental cost-effectiveness ratios (ICER) (e.g., cost per disability-adjusted life year [DALY] averted, cost per quality-adjusted life year [QALY] gained).

### Study selection procedure

The citations including title and abstracts were first assessed against the eligibility criteria by two independent reviewers (HM and SK). Where the applicability of the inclusion criteria was unclear, the article was included in the next stage to ensure that all potentially relevant studies were identified. Full-text copies of publications potentially meeting the eligibility criteria were then obtained and reviewed against the same eligibility criteria by two reviewers (HM and SK). At both the title/abstract and full-text review stages, any disagreements between the reviewers were resolved by discussion until a consensus was met, with a third independent reviewer making the final decision if necessary (MU). No language restrictions were applied.

### Data extraction

For full-text studies, data were extracted by a single reviewer (SK) into a pre-specified data extraction form and was independently checked for completeness and accuracy by a second reviewer (SN).

Extracted information included the following:

- Details of study context (authors, publication year, country, setting, study population, intervention and comparators).
- A detailed account of the economic evaluation methods and results (type of economic evaluation, model type, study perspective, time horizon, currency and price year, discount rate, resource use/costs, outcome measures, analytical methods, results, sensitivity analyses, generalisability, conclusion, source of funding and conflicts of interest).

### Quality appraisal of economic evaluations

To allow a comparison of the economic evaluation methods used in the studies, the reporting quality of both the trial-based and model-based economic evaluations were assessed using the CHEERS 2022 checklist [14] which is a commonly used generic quality assessment tool of reporting standards. The quality of each model-based economic evaluation was further assessed using the Philips checklist [15]. The quality assessment checklists provide a systematic and critical descriptive overview of key methodological elements. Quality assessment was undertaken by one reviewer (SK) and was independently checked for completeness and accuracy by a second reviewer (SN).

## Results

**Fig. 1:**
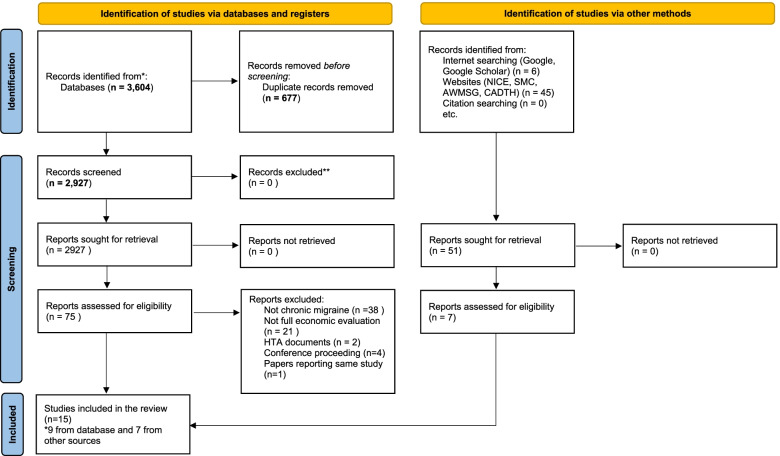
PRISMA flow chart diagram

After deduplication, we identified 2927 citations from the database searches and reviewed them at the title/abstract review stage. After title/abstract review, 75 articles were reviewed in the full-text stage and nine articles [16–24] ultimately met the inclusion criteria for published peer-reviewed journal articles. One article was translated from Bulgarian [24] and one article was translated from Italian [22]. Our translation is available on request from the corresponding author. We also found an additional 51 reports and after the screening, we were left with seven reports [25–31] which were identified through other methods, such as Google, Google Scholar and websites of NICE, SMC, AWMSG and CADTH that were not published in peer review journals. The flow of studies through the systematic review process is presented in Fig. 1. We have narratively synthesised the reporting of the nine published journal articles separately from the seven reports.

### Context, types of economic evaluation, interventions and comparators

Among the nine included studies from the database search, the majority of studies (*n* = 7) [17–21, 23, 24] were published during the last 5 years. All studies originated from high-income countries, mainly countries in European Union (*n* = 5) [17, 18, 21, 22, 24], including two each in the UK [16, 19] and the United States [20, 23]. All the evaluations were model-based cost-utility analyses involving hypothetical cohorts of the participants with chronic migraine [16–21, 23]. Among the nine included studies, four studies [16, 18, 19, 22] evaluated Botox and five studies [17, 20, 21, 23, 24] evaluated Erenumab for the treatment of chronic migraine. All the studies evaluating Botox [16, 18, 19, 22] compared the treatment with placebo or the best supportive care. As no established standard of care existed participants took their usual acute headache medications, placebo treatment (injection of saline water) was also considered as a comparator and assumed to represent consultant appointments to tailor acute medication, such as triptans. Those studies evaluating Erenumab compared the outcomes with Botox as a primary comparator or as a part of a sensitivity analysis. Further details about the interventions and comparators are presented in Table 1.

**Table 1:** Summary of the characteristics of included studies

Authors (Year), Country	Objective(s)	Study design	Study population	Sub-groups	Sample size (n)	Intervention	Comparators	Type of Economic Evaluation
**Journal Articles**								
Batty AJ, et al. (2013), United Kingdom [16]	To evaluate the cost-effectiveness of Ona botulinumtoxin A (Botox) compared with placebo for the prophylaxis of headaches in adults with chronic migraine (CM)	Model based economic evaluation	Participants in The Phase III REsearch Evaluating Migraine Prophylaxis Therapy (PREEMPT) Trial were considered for the model.	The groups comprised: 1) The licensed population, of all CM participants (*n* = 401), 2) Participants who have previously received one or more oral prophylactic treatments (only Topiramate was a licensed treatment for migraine) (*n* = 983), and 3) Participants who have previously received three or more oral prophylactic treatments (*n* = 439)	1384	Botox	Placebo	Cost-utility analysis (CUA)
Giannouchos TV, et al. (2019), Greece [17]	To evaluate the differences in costs and outcomes of the preventive treatment with Erenumab versus ONBTA in CM participants	Model based economic evaluation	Participants with CM who fail initial preventive treatment with ONBTA or Erenumab. Adults with a mean age 41 years; and 86% were females.	None	Not reported	Erenumab	Botox	CUA
Hansson-Hedblom A, et al. (2020), Norway and Sweden [18]	To describe the economic consequences of migraine in Sweden using cost of illness survey data and the cost-effectiveness of ONBTA for the treatment of CM	Model based economic evaluation	Participants in Phase III PREEMPT trial	As in other study using PREEMPT trial participants	Not reported	Botox	Placebo	CUA
Hollier-Hann G, et al. (2020), United Kingdom [19]	To evaluate the cost-effectiveness of Botox compared with placebo for the prophylaxis of headaches in adults with CM	Model based economic evaluation	Participants with CM who have previously received three or more oral preventive therapies in PREEMPT Trial	None	439	Botox	Placebo	CUA
Lipton RB, et al. (2018), United States of America [20]	To estimate value-based pricing ranges for Erenumab 140 mg, administered subcutaneously every 4 weeks, in participants who have failed at least 1 prior preventive treatment compared to supportive care (SC)	Model based economic evaluation	Participants that were either naïve to preventive treatment or previously treated with preventive medication but failed due to lack of efficacy or intolerability. The migraine populations considered in the model are sub-groups of participants who have previously failed “1 prior preventive therapy.	Chronic and episodic migraine (EM) group	Not reported	Erenumab	Placebo (vs Botox as a scenario analysis)	CUA
Mahon R, et al. (2021), Sweden [21]	To determine the cost-effectiveness of Erenumab for the preventive treatment of migraine.	Model based economic evaluation	Participants with CM and EM. The base-case analysis for ‘total migraine’ assumed that 66.7% of the participants had CM and 33.3% had EM, which aligns with the reported percentage of participants with CM for whom prophylactic treatment fails.	None	Not reported	Erenumab	Placebo (vs Botox as a scenario analysis)	CUA
Ruggeri et al. (2013), Italy [22]	To evaluate the cost-effectiveness of Botox versus placebo in participants with CM	Model based economic evaluation	Participants with CM from PREEMPT trial.	None	1384 participants. 686 received treatment and 698 received placebo	Botox	Placebo	CUA
Sussman M, et al. (2018), United States of America [23]	To assess the cost-effectiveness of Erenumab for the prophylactic treatment of EM and CM	Model based economic evaluation	Participant with EM and CM. The analyses were done separately	CM and EM groups	Not stated	Erenumab	Placebo (vs Botox as a scenario analysis)	CUA
Vekov (2019), Bulgaria [24]	To develop a model based on local data on costs and health benefits of alternative Calcitonin gene-related peptide (CGRP) inhibitors in Bulgaria	Model based economic evaluation	Participants with EM and CM	CM and EM groups. For the CM group only participants who have not improved with standard preventive therapy were included	667	Erenumab	Preventative treatment	CUA
**Other Reports**								
CADTH (Botox) (2019), Canada [25]	To compare cost-effectiveness of Botox with existing treatments	Canada	Model based economic evaluation	Participants with CM from PREEMPT trial.	1384	Botox	Best supportive care (BSC)	CUA
CADTH (Erenumab) (2019), Canada [26]	To compare cost-effectiveness of Erenumab with existing treatments	Canada	Model based economic evaluation	Adult participants with CM, defined as headache 15 or more days per month and headache lasting four hours a day or longer (Study 295, STRIVE trial and LIBERTY trial)	Not stated	Erenumab	BSC, (vs Botox in scenario analysis)	CUA
ICER (2018), United States of America [27]	To compare cost-effectiveness of Calcitonin Gene- Related Peptide (CGRP) inhibitors as the preventative treatments for participants with EM or CM	USA	Model based economic evaluation	Patients with CM who fail initial preventive treatment with Botox or other treatment for the prevention of migraine attack	Not stated	Erenumab, Fremanezumab	BSC	CUA
NICE: Erenumab (2019), United Kingdom [29]	To compare cost-effectiveness of Erenumab with existing treatments	UK	Model based economic evaluation	Patients with CM who fail initial preventive treatment with Botox or other treatment for the prevention of migraine attack.	439	Erenumab	BSC and Botox	CUA
NICE: Fremanezumab (2019), United Kingdom [28]	To compare cost-effectiveness of Fremanezumab with existing treatments	UK	Model based economic evaluation	Patients with CM who fail initial preventive treatment with Botox or other treatment for the prevention of migraine attack.	439	Fremanezumab	BSC and Botox	CUA
NICE: Galcenzenumab (2020), United Kingdom [30]	To compare cost-effectiveness of Glacanzunab with existing treatments	UK	Model based economic evaluation	Patients with CM who fail initial preventive treatment with Botox or other treatment for the prevention of migraine attack.	439	Glacanzunab	BSC and Botox	CUA
Warwick Evidence (2011), United Kingdom [31]	To compare cost-effectiveness of Botox with existing treatments	UK	Model based economic evaluation	Patients in The Phase III REsearch Evaluating Migraine Prophylaxis Therapy (PREEMPT) Trial were considered for the model.	1384	Botox	Placebo	CUA

*CGRP* Calcitonin gene-related peptide, *CM* Chronic migraine, *CUA* Cost utility analysis, *EM* Episodic migraine, *ONBTA* Ona botulinumtoxin A, *PREEMPT* Patients in The Phase III REsearch Evaluating Migraine Prophylaxis Therapy, *SC* Supportive care

In addition to the journal databases search, there were seven additional reports identified. Four reports were from the United Kingdom [28–31], two from Canada [25, 26] and one from the United States of America [27]. The reports evaluated the cost-effectiveness of Botox [25, 31], Erenumab [26, 27, 29], Fremanezumab [28], and Galcenzumab [28]. The treatments were compared either with placebo (best supportive care) [25, 27–31] or Botox [26, 28–30] and all of them were cost-utility analyses.

### Modelling approach, perspective and time horizon used in the models

All of the journal articles were cost-utility studies. The majority (*n* = 6) [16, 18–20, 22, 24] of studies utilised a Markov state-transition model structure. Markov models commonly included health states stratified by the number of migraine or headache days per 28 days and allowed for treatment discontinuation or treatment on/off periods. One study used a decision tree, and the other two studies were based on the hybrid models: the first, a hybrid of decision tree and Markov model and the second, a hybrid of a Monte Carlo simulation and Markov model. The time horizon most utilised was two-years, however, the length varied from 1 year to 10 years. The studies from the United Kingdom were conducted from the National Health Service (NHS) perspective, the European studies were based on the societal perspective and the American studies were based on societal and payers’ perspectives.

Examination of the reports suggested two different types of model being implemented: Markov model with health states stratified by number of headache days per 28 days as mentioned above [31] or hybrid model with decision tree for 12-week assessment period, classifying participants as responders and non-responders, and Markov model for post-assessment with 12-week cycle lengths [25–30]. There were two studies which used a lifetime horizon (25 years) [29, 30] and the rest of them used a time horizon ranging from two to 10 years [25–28, 31]. Further details about the modelling approach, perspective and time horizon used in the models are presented in Table 2.

**Table 2:** Details of the Models and Model Inputs in the studies included in the Systematic Review

Authors (Year), Countries	Model type	Perspective	Time horizon	Cost included in the model	Source of cost and resource inputs	Currency, price year
**Journal Articles**						
Batty AJ, et al. (2013), United Kingdom [16]	A Markov model with 13 health states, including death. The 12 states were split into two parallel stages: on treatment and off treatment. A 12-weeks cycle length was employed. The model also considered negative and positive stopping rule for the treatment.	National Health Service (NHS)	2 years	Cost of Botox; Consultant time to take participant history, tailor prophylactic and acute treatment; consultant time to administer the injections; cost of care including general practitioner (GP) visits, emergency department (ED) visits, hospitalisation and triptan costs.	Resource used was informed by International Burden of Migraine Study (IBMS), with unit costs taken from NHS reference cost, cost of triptans per attack was based on the weighted average costs in the UK in 2010.	UK £ 2010
Giannouchos TV, et al. (2019), Greece [17]	Decision tree model.	Payer and Societal perspective	1 year	Direct costs included the cost of the two drugs and administration, the use of acute drugs under usual care, and hospitalisation costs, physician, and ED visits. Indirect costs for the societal perspective analysis included wages lost on workdays.	Resource utilisation data were obtained from four previously published studies and the cost inputs were obtained from publicly available data for the Greek healthcare sector and on the governmental pricing system derived from a public Greek hospital.	Euro € 2019
Hansson-Hedblom A, et al. (2020), Norway and Sweden [18]	A Markov model with 13 health states, including death as mentioned in Batty AJ, et al.	Payer and Societal perspective	10 years	Direct cost included cost of Botox, Neurology consultant appointment, specialist nurse appointment, cost of care including GP visits, ED visits, hospitalisation and triptan costs. Indirect cost involved productivity cost.		SEK, 2018 for Sweden; NOK, 2018 for Norway
Hollier-Hann G, et al. (2020), United Kingdom [19]	CUA using Markov model with 13 health states, including death as mentioned in Batty AJ, et al.	NHS	2 years	Cost of Botox; Consultant time to take participant history, tailor prophylactic and acute treatment; consultant time to administer the injections; cost of care including GP visits, ED visits, hospitalisation and triptan costs.	Resource used was informed by IBMS, with unit costs taken from NHS reference cost, cost of triptans per attack was based on the weighted average costs in the UK in 2010.	UK £ 2010
Lipton RB, et al. (2018), United States of America [20]	A Markov model was implemented based on the clinical data from the Episodic Migraine (EM) and Chronic Migraine (CM) studies for the sub-groups of participants with prior treatment failures. The cycle length was 28 days.	US societal perspective	10 years	Direct medical costs included cost of medicine and its administration, GP visits, ED visits, hospitalisations, and specialist neurologist consultations based on published unit costs. Cost of medicines to treat acute attacks. Indirect costs included productivity cost associated with presentism and absenteeism.	Average annual medical resource use is taken from a published 2009 analysis of survey data from 7437 migraine participants in the US	USD $ 2017
Mahon R, et al. (2021), Sweden [21]	A hybrid decision-tree plus Markov model was developed	Swedish societal perspective	10 years	Direct cost included cost of medicine and its administration, ED visit, hospitalisation, GP visit, consultant visit, Nurse/physician visit, Triptan medication and other medications. Indirect cost related to absenteeism and presenteeism were included.	Resource utilisation and efficacy data were sourced from four trials (CM295, STRIVE, ARISE and LIBERTY). Study 178, which had an open-label phase of 256 weeks, was used to inform long-term assumptions regarding those who continued treatment. Resource usage cost were obtained from the price list of Southern Sweden. Productivity costs were included from the published literature.	SEK, 2018
Ruggeri et al. (2013), Italy [22]	A Markov model with 13 health states, including death as mentioned in Batty AJ, et al.	Italian National Health Service and a societal perspective.	2 years	Direct cost included cost of medicine and its administration, GP visit or outpatient cost, ED visit, hospitalisation and cost of Triptans. Indirect costs included productivity cost.	Resource utilisation data was derived from IBMS study. Costs were obtained from the local government data.	Euro € 2013
Sussman M, et al. (2018), United States of America [23]	A hybrid Monte Carlo participant simulation and Markov cohort model was constructed. Participants in both cohorts (EM and CM) must have failed at least one previous preventive therapy prior to model entry since Calcitonin gene-related peptide (CGRP) pathway antagonists are expected to be used as second-line therapies. Participants in the EM cohort must have had between 4 and 14 monthly migraine days (MMDs) and participants in the CM cohort must have had at least 15 MMDs at baseline.	US Societal and Payers perspective	2 years	Direct cost included - acute medication cost, physician visit, ED visit, adverse events, and hospitalisation cost. Indirect costs included productivity cost	Data inputs for the model were derived from the Erenumab pivotal and Open labelled extended trials, and Botox pivotal trial, published literature, and publicly available sources.	USD $ 2017
Vekov (2019), Bulgaria [24]	A hybrid model including a Monte Carlo simulation and a Markov cohort model. The input data to the model are the primary and secondary clinical endpoints in the randomized trials NCT02066415 and NCT02483585. They measure the change in the number of days with migraine per month at weeks 12 and 24, the number of days per month with symptomatic migraine therapy	Payers’ perspective	2 years	Only the cost of medicines was included; other healthcare costs were assumed to be equal for both therapies and hence excluded.	Resource utilisation (medicine usage) data was obtained from the randomised trial NCT02066415.	Bulgarian Rev. (BGN) 2019
**Other reports**						
CADTH (Botox) (2019), Canada [25]	Hybrid model with decision tree for 12-week assessment period, classifying patients as responders and non-responders, and Markov model for post-assessment with 12-week cycle lengths.	Canadian public health care payer perspective	3 years	Direct costs included cost of medicine and its administration, GP visits or outpatient cost, ED visits, hospitalisation and cost of Triptans. Indirect costs included productivity cost.	Resource used was informed by IBMS, with unit costs taken from NHS reference costs, cost of triptans per attack was based on the weighted average costs in the UK in 2010.	CAD $ 2019
CADTH (Erenumab) (2019), Canada [26]	Hybrid model with decision tree for 12-week assessment period, classifying participants as responders and non-responders, and Markov model for post-assessment with 12-week cycle lengths.	Canadian public health care payer perspective	3 years	Direct costs included cost of medicine and its administration, GP visits or outpatient cost, ED visits, hospitalisation and cost of Triptans. Indirect costs included productivity cost.	Resource used informed by the trial and cost data was obtained from manufacturer and other local data resources	USD $ 2018
ICER (2018), United States of America [27]	Markov model comprising CGRP inhibitor versus no preventive treatment arms. The intervention arm of the model includes three health states: 1) CGRP inhibitor treatment, 2) no preventive treatment, and 3) death. The comparator arm includes two health states: 1) no preventive treatment and 2) death.	Health system payer perspective	2 years	Direct medical care cost including cost of medicine, GP visit, outpatient visit cost, ED visit and hospitalisation.	Resource used was informed by International Burden of Migraine Study (IBMS), with unit costs taken from the local data resources	CAD $ 2019
NICE: Erenumab (2019), United Kingdom [29]	A decision-tree plus Markov model included two health states - on treatment and discontinuation of treatment once patients were classified as responders or non-responders.	NHS perspective	Lifetime	Migraine specific cost related to hospitalisation and ED visits, health care professional visits and use of acute medication.	Resource used was informed by National Health and Wellness survey conducted in migraine population, with unit costs taken from the local data resources	UK £ 2018
NICE: Fremanezumab (2019), United Kingdom [28]	A decision-tree plus Markov model included two health states - on treatment and discontinuation of treatment once patients were classified as responders or non-responders.	NHS perspective	10 years	Migraine specific cost related to hospitalisation and ED visits, health care professional visits and use of acute medication.	Resource used was informed by National Health and Wellness survey conducted in migraine population, with unit costs taken from the local data resources	UK £ 2019
NICE: Galcenzenumab (2020), United Kingdom [30]	A decision-tree plus Markov model included two health states - on treatment and discontinuation of treatment once patients were classified as responders or non-responders.	NHS perspective	Lifetime	Migraine specific cost related to hospitalisation and ED visits, health care professional visits and use of acute medication.	Trial-specific (CONQUER) data and the resource utilisation data from Lipton et al. (2018) [20].	UK £ 2020
Warwick Evidence (2011), United Kingdom [31]	A Markov model with 13 health states, including death. The 12 states were split into two parallel stages: on treatment and off treatment. Each treatment state was sub-divided into categories based on the number of headache days per 28 days. Three health states for EM (0–3, 4–9, and 10–14 headache days per 28 days), and three health states for CM (15–19, 20–23, and 24+ headache days per 28 days). A 12-weeks cycle length was employed. The model also considered negative and positive stopping rule for the treatment.	NHS perspective	2 years	Migraine specific cost related to hospitalisation and ED visits, health care professional visit and use of acute medication.	Resource used was informed by IBMS, with unit costs taken from NHS reference cost, cost of triptans per attack was based on the weighted average costs in the UK in 2010.	UK £ 2011

*CGRP* Calcitonin gene-related peptide, *CM* Chronic migraine, *CUA* Cost utility analysis, *ED* Emergency department, *EM* Episodic migraine, *GP* General practitioner, *IBMS* International Burden of Migraine Study, *MMD* Monthly migraine days, *NHS* National Health Service

### Resource utilisation and cost

In most of the studies published in the journal articles, the cost included the cost of pharmacotherapy and healthcare resource utilisation. Direct costs included the cost of the medicine and the administration costs, the costs of acute drugs used under usual care, and the costs of hospitalisation, physician, neurology appointment, specialist nurse and/or emergency department visits. Indirect costs for the societal perspective analyses included wages lost on workdays. In some studies (*n* = 4), the resource use data were obtained from the International Burden of Migraine Studies (IBMS) [16–19, 22, 24] and for other studies, they were either obtained from other published studies or local databases. The NHS reference costs were used as the source of cost inputs in the UK studies [16, 19], whereas other studies relied on other specific trials or publicly available sources. All studies included in the reports [25–31] also included similar resource usage data and unit cost data. The summary of the resource utilisation and the associated cost is illustrated in Table 2.

### Price year/currency, the discount rate used and willingness-to-pay (WTP threshold)

All studies published in the journal articles reported the currency and price year. The UK studies [16, 19] used a 3.5% discount rate for costs and benefits, all other European studies [17, 18, 21, 22], except one [24] and the American studies [20, 23] applied a discount rate of 3% for both costs and outcomes. The studies from the UK [16, 19] used £20,000– £30,000/QALY as the willingness-to-pay (WTP) threshold. The studies from the United States used different WTP thresholds, for example, Lipton et al. (2018) [20] used $100,000–$200,000/QALY and Sussman et al. (2018) used $50,000/QALY thresholds [23]. Among the European studies, Giannouchos et al. (2019) [17] used Euro 49,000/QALY, Ruggeri et al. (2013) [22] used Euro 20,000- 30,000, Hansson-Hedblom A, et al. (2020) [18] used SEK 280,000 for Sweden and NOK 495,000 for Norway, and Mahon et al. (2021) [21] used SEK 300,000/QALY as the WTP thresholds. The study from Bulgaria [24] used 3 times their GDP per capita as the WTP threshold.

As in the journal articles, the studies for the reports also reported the currency and price year. The studies from UK used a 3.5% discount rate for costs and benefits [28–31], the Canadian [28–31] and American studies used a 3% discount rate. Further details on price year/currency, the discount rate used and the WTP thresholds are illustrated in Table 3.

**Table 3:** Model inputs (continued), Results and Sensitivity Analyses reported in the included studies

Authors (Year), Countries	Discount rate	Utilities (QALYs) and outcomes			Results/ICER	Willingness-to-pay threshold	Sensitivity analyses
		Preference based measure used to estimate utilities	Whose utility values?	Other outcomes			
**Journal Articles**							
Batty AJ, et al. (2013), United Kingdom [16]	3.5%	Migraine Specific Quality of Life Questionnaire v2.1 (MSQ) was used to collect Health-Related Quality of Life (HRQoL) information at baseline and 24 weeks after the intervention. The MSQ scores were mapped to EQ-5D to produce utility values.	Utility values from the participants of PREEMPT trial	Headache per day/year, cost per headache day avoided	At 2 years, Botox treatment was associated with an increase in costs of £1367 and an increase in QALYs of 0.1 compared to placebo, resulting in an incremental cost-effectiveness ratio (ICER) of £15,028. Treatment with Botox reduced headache days by 38 days per year at a cost of £18 per headache day avoided.	£20,000– £30,000/QALY	Both deterministic and probabilistic sensitivity analyses (PSA) were performed
Giannouchos TV, et al. (2019), Greece [17]	None	Quality-adjusted life years (QALYs) were calculated by using the health utility data (MSQ to EQ-5D) for participants with chronic migraine (CM) from 10 countries obtained from the International Burden of Migraine Study (IBMS)	General public	Number of migraines avoided	CM treatment with Erenumab compared to Botox resulted in ICERs of €218,870 and €231,554 per QALY gained and €620 and €656 per migraine avoided, from the societal and the payer’s perspective, respectively. Using a cost-effectiveness threshold equal to three times the local gross domestic product (GDP) per capita (€49,000), for Erenumab the ICERs fall below this threshold.	EURO 49,000/QALY	Both PSA and deterministic sensitivity analyses were performed
Hansson-Hedblom A, et al. (2020), Norway and Sweden [18]	3%	The IBMS study was used to map EQ-5D scores from MSQ score.	Utility values from the participants of PREEMPT trial		In Sweden, Botox was associated with 0.223 additional QALYs at an additional cost of EUR 4126 compared to placebo, resulting in an ICER of EUR 18,506. In Norway, Botox was associated with 0.216 additional QALYs at an additional cost of EUR 4301 compared to placebo, resulting in an ICER of EUR 19,954.	SEK 280,000 (Sweden) and NOK 495,000 (Norway)	Both PSA and deterministic sensitivity analyses were performed
Hollier-Hann G, et al. (2020), United Kingdom [19]	3.5%	Utility values were directly obtained from the EQ-5D data collected in the REPOSE study. EQ-5D was administered at baseline and each follow-up visit (at intervals of approx. 12 weeks)	UK tariff	Headache per day/year, cost per headache day avoided	Botox treatment resulted in incremental costs of £1204 and an incremental QALY gain of 0.07 compared with placebo in CM participants who have previously failed three or more preventive treatments, corresponding to an ICER of £16,306 per QALY gained	£20,000– £30,000/QALY	Both PSA and deterministic sensitivity analyses were performed
Lipton RB, et al. (2018), United States of America [20]	3%	MSQ responses from the Erenumab EM and CM pivotal studies were mapped to the EQ-5D-3L, then pooled to generate one complete migraine dataset.	General public		Erenumab resulted in incremental QALYs of 0.185 vs supportive care (SC) and estimated cost offsets due to reduced monthly migraine days (MMD) of $8482 over 10 years, with an average duration of treatment of 2 years	$100,000–$200,000/ QALY	Both PSA and deterministic sensitivity analyses were performed
Mahon R, et al. (2021), Sweden [21]	3%	Two trials included in this study used the MSQ score which was mapped onto EQ-5D.	Not stated	Cost per migraine day avoided	Erenumab treatment resulted in ICERs of Swedish krona (SEK) 34,696 and SEK 301,565 per QALY gained in the total migraine and episodic migraine (EM) populations, respectively. Erenumab was dominant in the CM population.	SEK 300,000/ QALY	Both PSA and deterministic sensitivity analyses were performed
Ruggeri et al. (2013), Italy [22]	3%	The IBMS study was used to map EQ-5D scores from MSQ score	UK tariff	Headache per day/year, cost per headache day avoided	Botox compared with placebo gained an incremental 0.04 more QALYs per participant; the incremental cost per participant was €208; the ICER was €4899 per QALY gained	€20,000 - €30, 000/QALY	Both PSA and deterministic sensitivity analyses were performed
Sussman M, et al. (2018), United States of America [23]	3%	EQ-5D scores were used	Not stated	Headache-related disability, lost work productivity, anxiety and depression	From a societal perspective treatment with Erenumab compared with no preventive treatment ranges from a dominant strategy among CM participants to an ICER of $122,167 for EM participants. When excluding indirect costs (i.e., payer perspective), the ICERs are cost-effective among CM participants ($23,079 and $65,720 versus no preventive treatment and Botox, respectively), but not among EM participants.	USD 50,000/ QALY	Both PSA and deterministic sensitivity analyses were performed
Vekov (2019), Bulgaria [24]	5%	EQ-5D scores were used	Not stated	Headache Impact Test (HIT-6), Migraine Disability Assessment, (MIDAS)	Erenumab was not cost-effective compared to placebo (standard prevention therapy) with ICER of 637,000 BGN/QALY.	Three times the national annual GDP per capita	PSA
**Other reports**							
CADTH (Botox) (2019, Canada [25]	3%	MSQ was used to collect HRQoL information at baseline and 24 weeks after the intervention. The MSQ scores then were mapped into to EQ-5D to produce utility values.	Utility values from the participants of PREEMPT trial were used	Headache per day/year, cost per headache day avoided	ICER was CAD 134,601/QALY gained for Botox vs BSC. At a WTP of CAD 50,000 per QALY, Botox was associated with a 9% probability of being the optimal intervention. A price reduction of more than 75% is required to achieve an ICER of less than CAD 50,000/QALY.	CAD 50,000	Sensitivity analysis showed that utility values had the greatest influence on model results.
CADTH (Erenumab) (2019, Canada [26]	3%	MSQ was used to collect HRQoL information at baseline and 24 weeks after the intervention. The MSQ scores then were mapped into to EQ-5D to produce utility values.	Utility values from the participants of PREEMPT trial were used	Headache per day/ year, cost per headache day avoided	Erenumab dominated Botox in the population for whom the previous treatment including Botox was failed.	CAD 50,000	Sensitivity analyses involved analysing different time horizon and with Scenarios were performed.
ICER (2018), United States of America [27]	3%	MSQ was used to collect HRQoL information at baseline and 24 weeks after the intervention. The MSQ scores then were mapped into to EQ-5D to produce utility values.	Utility values from the participants of PREEMPT trial were used	Headache per day/year, cost per headache day avoided	The ICER for Erenumab vs no preventative treatment was USD 86,000/QALY and Fremanzenumab vs no preventative treatment was USD 115,000/QALY, both way above the baseline WTP of USD 50,000/QALY.	USD 50,000	Sensitivity analyses were performed using topiramate as the alternative treatment to Botox and this resulted in an estimated ICER of USD 28,960/QALY.
NICE: Erenumab (2019), United Kingdom [29]	3.5%	MSQ was used to collect HRQoL information at baseline and 24 weeks after the intervention. The MSQ scores then were mapped into to EQ-5D to produce utility values.	Utility values obtained from Erenumab trials (Study 295, STRIVE and ARISE) data.	Headache per day/year, cost per headache day avoided	The blended dose of Erenumab was cost-effective in treating CM population vs Botox and vs best supportive care with a ICER of £18,893 and an ICER of £17,212 per QALY gained, respectively. Erenumab 140 mg is cost-effective treatment vs both Botox and best supportive care, with an ICER of £17,832 and an ICER of £13,340 per QALY gained, respectively.	£20,000– £30,000/QALY	Both PSA and deterministic sensitivity analyses were performed including using the whole migraine population and a societal perspective.
NICE: Fremenzumab (2019), United Kingdom [28]	3.5%	MSQ was used to collect HRQoL information. The MSQ scores then were mapped into to EQ-5D to produce utility values.	Utility values obtained from patient level MSQ data from FOCUS trial	Headache per day/year, cost per headache day avoided	Fremanezumab had higher costs, but also gained more QALYs than both best supportive care and Botox. The ICERs showed that Fremanezumab was a cost-effective treatment compared to best supportive care (£11,825/QALY gained) and Botox (£16,227/QALY gained)	£20,000– £30,000/QALY	Both PSA and deterministic sensitivity analyses were performed
NICE: Galcenzenumab (2020), United Kingdom [30]	3.5%	MSQ was used to collect HRQoL information. The MSQ scores then were mapped into to EQ-5D to produce utility values.	Utility values obtained from patient level MSQ data from CONQUER trial	Headache per day/ year, cost per headache day avoided	The actual ICERS were confidential and masked. However, the report indicated that ICER for Galcanezumab fall below the lower threshold (£20,000/QALY gained) as defined by standard WTP for United Kingdom.	£20,000– £30,000/QALY	Both PSA and deterministic sensitivity analyses were performed
Warwick Evidence (2011), United Kingdom [31]	3.5%	MSQ was used to collect HRQoL information. The MSQ scores then were mapped into to EQ-5D to produce utility values.	Utility values obtained from patient level MSQ data from PREEMPT Trial	Headache per day/year, cost per headache day avoided	The reported ICER was £5828/QALY gained.	£20,000– £30,000/QALY	Both PSA and deterministic sensitivity analyses were performed

*CM* Chronic migraine, *EM* Episodic migraine, *EQ-5D* European-Quality of Life Five dimensions, *HRQoL* Health-Related Quality of Life, *GDP* Gross domestic product, *IBMS* International Burden of Migraine Study, *ICER* Incremental cost-effectiveness ratio, *HIT-6* Headache Impact Test, *MIDAS* Migraine Disability Assessment, *MSQ* Migraine specific questionnaire, *MMD* Monthly migraine days, *NOK* Norwegian Krone, *PREEMPT* Patients in The Phase III REsearch Evaluating Migraine Prophylaxis Therapy, *PSA* Probabilistic sensitivity analyses, *QALY* Quality-adjusted life year, *SEK* Swedish Krona, *SC* Supportive care

### Utilities and outcomes

The 5-level EQ-5D (EQ-5D-5L) was the main outcome measure used in the economic models. Except for Mahon et al. (2020) [21] study, which used the 3-level EQ-5D (EQ-5D-3L) measure. In four cases [16, 17, 20, 21], the EQ-5D scores were mapped from the Migraine Specific Questionnaire (MSQ). Whereas Sussman et al. (2018) used EQ-5D-5L scores from Erenumab pivotal and open labelled trials (OLE) and Botox pivotal trials [23]. In the case of Hollier-Hann, et al. (2020) [19], utility values were directly obtained from the EQ-5D-5L data collected in the REPOSE study. The EQ-5D-5L was administered at baseline and each follow-up visit (at intervals of approx. 12 weeks) in participants receiving Botox. EQ-5D scores were categorised according to the number of headache days as per the health states in the model and utilities were calculated using the UK value set. For each health state in the Markov model, the mean migraine days (MMD)/28 days specific utilities values were assigned. All the studies in the reports [25–31] also used EQ-5D-5L as the main outcome measure. Other outcomes reported by the studies included headache day/year, cost per headache day avoided, cost-saving associated with the use of medicine, headache-related disability, and lost work productivity (Table 3).

### A narrative synthesis of cost-effectiveness evidence

In all journal studies which used Botox as the treatment, this was associated with a favourable incremental cost-effectiveness ratio (ICER). The use of Botox was cost-effective compared to placebo, with an ICER ranging between £15,028 (€17,720) and £16,598 (€19,572) [16, 18, 19, 22]. For example, in the study by Batty et al. [16], an increase in costs of £1367 and an increase in QALYs of 0.1 compared to placebo, resulted in an ICER of £15,028. Treatment with Botox reduced headache days by an estimated 38 days per year at the cost of £18 per headache day avoided. Although the figures for ICERs may be different, in all countries Botox was judged to be cost-effective in their widely accepted respective WTP thresholds. Erenumab, was also found to be cost-effective in participants for whom previous preventive treatments did not work. Two studies also compared the cost-effectiveness of Erenumab against the placebo and found that Erenumab was dominant. For example, Sussman et al. (2021) [23] reported that with an annual drug price of Erenumab at $6900, from a societal perspective treatment with Erenumab is dominant (where it is cheaper and more effective) compared with no preventive treatment among chronic migraine participants. When excluding indirect costs (i.e., payer perspective), the ICERs are cost-effective among chronic migraine participants ($23,079 and $65,720 versus no preventive treatment and Botox, respectively [23]. When Erenumab was used as the treatment compared to Botox, the ICERs ranged between £59,712 ($81,080) [20] and £182,128 (€218,870) [17], with the ICERs above the most common WTP thresholds.

From the reports, all CGRP inhibitors like Erenumab, Galcanezumab and Fremanezumab were found to be cost-effective in the chronic migraine population for whom the previous three treatments did not work under the widely accepted WTP thresholds [26–30] and have been recommended for such group of participants. More information about the cost-effectiveness of the treatments is provided in Table 3.

### Sensitivity analyses

All of the studies were robustly analysed by using deterministic and/or probabilistic sensitivity analyses (PSAs). In most cases, the results were sensitive to changes in monthly migraine days, health utilities, and treatment costs but were cost-effective overall. For example, Hollier-Hann et al. (2020) [19] showed that the administration of Botox by a specialist nurse rather than a neurology consultant reduced the ICER from £16,306 to £13,832 per QALY gained and removal of the positive stopping rule recommended in current NICE guidance increased the ICER to £20,768 per QALY for Botox vs. placebo. Combining these two scenarios produced an ICER of £17,686 per QALY gained. To explore parameter uncertainty, Hollier-Hann et al. (2020) [19] a PSA with 5000 simulations on all model inputs except for drug costs was conducted, generating an ICER of £16,738 which was relatively close to the deterministic ICER of £16,306. Botox had a 71.9% and 94.2% chance of being cost-effective at a threshold of £20,000 and £30,000 per QALY gained respectively. The sensitivity analyses performed in each of the reports were more comprehensive than the journal articles. Further information about the sensitivity analyses is presented in Table 3.

### Generalisability

To assess the level of generalisability, all studies published in peer-reviewed journals were classified as: [1] generalisable [2]; transferable; and [3] context-specific. Three studies [16, 19, 22] were transferable, and six studies [17, 18, 20, 21, 23, 24] were considered to be context-specific. The source of funding was disclosed in all articles, apart from two studies [17, 24]. Eight out of nine studies [16–23] reported if they had any conflicts of interest.

All of the reports were considered to be context-specific and none of them declared any conflicts of interest, although they were funded by the pharmaceutical industries except one report [31].

The journal articles and reports also mentioned if there were any limitations with their studies, further details are shown in Table 4.

**Table 4:** Other details about the included studies

Author	Limitations	Generalisability	Source of funding	Conflicts of interest
**Journal articles**				
Batty AJ, et al. (2013), United Kingdom [16]	Placebo was not representative of standard care	Transferable	Allergan Inc., Irvine, CA.	Stated
Giannouchos TV, et al. (2019), Greece [17]	Limitations were mostly presented for the assumptions made in the model.	Context-specific	None	Stated
Hansson-Hedblom A, et al. (2020), Norway and Sweden [18]	The clinical trial may not be representative of everyday practice and physicians and participants may adjust treatment practices. The model was limited by only using MMD, and other dimensions of migraine, such as duration and severity, were not considered.	Context-specific	Allergan Norden, AB.	Stated
Hollier-Hann G, et al. (2020), United Kingdom [19]	Limitations included the assumptions made for the model including that treatment response, HRQoL and resource utilisation were based on MMD frequency alone.	Transferable	Allergan UK, Marlow, Buckinghamshire, UK	Stated
Lipton RB, et al. (2018), United States of America [20]	The model was created based on primary efficacy data from a mixed population of participants (EM and CM). There was also limited data beyond week 12 for CM participants. Also, treatment response, HRQoL and resource utilisation were based on MMD frequency alone..	Context-specific	Amgen Inc.	Stated
Mahon R, et al. (2021), Sweden [21]	Limitations included the assumptions made for the model including that treatment response, HRQoL and resource utilisation were based on MMD frequency alone.	Context-specific	Novartis Pharma AG	Stated
Ruggeri et al. (2013), Italy [22]	Same limitations as Lipton, et al. (see above) and also the study used the UK tariff for the utility scores in the base model.	Transferable	Not stated	Stated
Sussman M, et al. (2018), United States of America [23]	Same limitations as Lipton, et al. (see above)	Context-specific	Amgen Inc.	Stated
Vekov (2019), Bulgaria [24]	Limitations were not stated	Context-specific	Not stated	Not stated
**Reports**				
CADTH (Botox) (2019, Canada [25]	The severity of CM was not captured in the model and there was no good quality of comparative evidence.	Context-specific	Allergan	None
CADTH (Erenumab) (2019, Canada [26]	There was no good quality of comparative evidence.	Context-specific	Novartis Pharmaceuticals Canada, Inc.	None
ICER (2018), United States of America [27]	Since the data was obtained from the trial, there was uncertainty about the long-term effectiveness of medicines.	Context-specific	Government and not-for-profit organisations	None
NICE: Erenumab (2019), United Kingdom [29]	Uncertainty due to not having long-term effectiveness data	Context-specific	Novartic Pharmaceutical UK Ltd.	None
NICE: Fremanezumab (2019), United Kingdom [28]	Uncertainty due to not having long-term effectiveness data. There was also a lack of granularity within the published data for Botox, which led to limitations within the network meta-analysis conducted to compare the efficacy of Fremanezumab and Botox.	Context-specific	Teva UK Limited.	None
NICE: Galcenzenumab (2020), United Kingdom [30]	The limitations included the model’s inability to capture the natural progression of diseases, the use of short-term estimates of mean change in monthly headache days, and response rates for extrapolating to different time horizons.	Context-specific	Eli Lilly and Company Limited	None
Warwick Evidence (2011), United Kingdom [31]	Limitations including limitation of the trial to deal with correlated data, predicted ED-5D scores and the integrity around utility scores.	Context-specific	NIHR, UK	None

*CM* Chronic migraine, *EM* Episodic migraine, *EQ-5D* European-Quality of Life Five dimensions, *HRQoL* Health-Related Quality of Life, *MMD* Monthly migraine days

### Quality appraisal of economic evaluations

The summary of the quality assessment results is presented Table 5. The quality of both the trial-based and model-based economic evaluations was assessed using the Consolidated Health Economic Evaluation Reporting Standards (CHEERS) 2022 checklist [14]. None of the included studies fulfilled all of the quality criteria. The majority of studies fulfilled a large number of quality criteria. The criteria that were the least well-addressed were the items on heterogeneity and generalisability. The quality of the model-based economic evaluations was further assessed using the Philips checklist [15]. Again, the majority of the studies fulfilled a large number of the quality criteria according to the Phillips checklist. The criteria that were least well addressed were whether the data has been assessed appropriately, the principles of uncertainty, heterogeneity, assumption about the continuity of treatment and its effect, including sensitivity analysis around the assumption of different alternatives of treatment effect.

**Table 5:** Number of quality assessment criteria addressed by individual studies

**Journal articles**									
	**Batty AJ, et al**	**Giannouchos TV, et al**	**Hansson-Hedblom A, et al**	**Hollier-Hann G, et al**	**Lipton RB, et al**	**Mahon R, et al**	**Ruggeri, et al.**	**Sussman M, et al**	**Vekov, et al**
**CHEERS 2022 (*n*** **= 28)**									
Yes	25	23	23	23	22	25	23	25	12
No	2	2	2	3	2	2	2	2	10
Partial	1	3	3	2	4	1	3	1	6
Unclear	0	0	0	0	0	0	0	0	0
Not applicable	0	0	0	0	0	0	0	0	0
**PHILIPS CRITERIA (*n*** **= 57)**									
Yes	51	50	50	49	51	51	50	51	20
No	3	4	4	6	4	3	3	3	16
Partial	3	3	2	2	2	2	2	3	9
Unclear	0	0	0	0	0	0	1	0	10
Not applicable	0	0	1	0	0	1	1	0	2
**Other reports**									
	**CADTH (Botox)**	**CADTH (Erenumab)**	**ICER**	**NICE (Erenumab)**	**NICE (Fremanezumab)**	**NICE (Galcenzenumab)**	**Warwick Evidence (Botox)**		
**CHEERS 2022 (*n*** **= 27)**									
Yes	27	26	25	27	27	27	24		
No	1	1	1	1	1	1	1		
Partial	0	1	2	0	0	0	3		
Unclear	0	0	0	0	0	0	0		
Not applicable	0	0	0	0	0	0	0		
**PHILIPS CRITERIA (*n*** **= 57)**									
Yes	50	49	52	53	54	55	55		
No	3	2	1	2	1	0	0		
Partial	4	6	4	1	1	1	2		
Unclear	0	0	0	1	1	1	0		
Not applicable	0	0	0	0	0	0	0		

## Discussion

Evidence on the cost-effectiveness of pharmacotherapies for the treatment of chronic migraine was systematically assessed in this review and we identified nine studies that were published in peer-reviewed journals and seven reports [16–24]. These studies evaluated four different medicines (Botox, Erenumab, Fremanezumab and Galcanezumab) and were generally classed as high quality when appraised by the CHEERS reporting tool. There has only been one recent review which was published in 2020 assessing the cost-effectiveness of the treatment for migraine including chronic and episodic migraines, however, this was limited to articles published from the UK and the Ireland [12]. Our review provides worldwide evidence and is more comprehensive than this published review. The 2020 review included eight studies and consisted of expert consultations that provided six recommendations on the ideal model structure for migraine that is clinically and economically meaningful. A decision-tree plus Markov structure was then developed for migraine therapies and was recommended for use in future studies.

In our review, all the economic evaluations were model-based, and the earlier economic evaluations used Markov models with health states based on the number of headache frequency per 28 days, and the latter economic evaluations used a hybrid model comprising decision-tree plus Markov model approach, including two health states of on treatment and discontinuation once patients were classified as responders or non-responders. Although the time horizons used by the earlier models ranged from 1 to 10 years, the more recent models used a lifetime horizon. There must be some agreement on this to use in the base-case model to help design future economic evaluations. Additionally, when a societal perspective was adopted, the costs attributed to presentism and absenteeism were far higher than the direct costs. In the earlier Markov models, there was stop/discontinue rules, and these rules were attributed to the fact that medicines can be discontinued in two situations, i.e., in case of when medicines worked or when they do not work (e.g., adverse drug reactions). Any future economic evaluations of chronic migraine treatments should consider such discontinuing rules in the model.

The burden that participants experience each day of migraine is meaningful. In a large retrospective, European cross-sectional study, an increase of one headache-free day (HFD) was associated with an average decrease in absenteeism of 3.9% and presenteeism of 2.1% [32]. Resource utilisation was also reduced, with an increase of one HFD being associated with expected decreases in healthcare provider and neurologist visits of 1.0% and 4.7%, respectively. The benefits of each additional HFD corresponded to average increases of 0.003 and 0.008 points for the SF-6D utility score and EQ-5D index score, respectively (*p* < 0.001 for all) [32]. The burden of migraine is worse for people who need to change preventive therapy. For these patients, 87% reported that migraine had a negative impact on professional, private, or social domains of life [33]. New therapies for chronic migraines, particularly those for treatment-refractory patients, can bring meaningful improvement for participants, and economic evaluations can be useful tools for assessing their value. If comparative data are available, the economic evaluation should include the ability to compare the treatment of interest with other preventive treatments. Compared to other disease areas, the number of economic evaluations in chronic migraine was limited. Many of these evaluations utilised a similar model structure, but each approach has unique strengths and limitations.

The main strength of this review is that it included the latest treatments for chronic migraine such as calcitonin gene-related peptides (CGRP) namely Erenumab, Fremanezumab and Galcanezumab as these have recently been approved for the treatment of chronic migraine. In spite of the worry about cost, these newer and better-tolerated CRGP antagonists treatments are cost-effective despite them being much more expensive than oral preventatives. Another strength of this review is the comprehensiveness of the search strategy used. The search was performed in a broad range of electronic databases of published studies. Furthermore, there were no country and language restrictions. The review also had some limitations. We only included full economic evaluations. Therefore, some important data contained within partial economic evaluations might have been missed. Another limitation of the study includes the unclear definition of comparators, such as best supportive care, placebo and preventative treatment within the included studies. But there was a general agreement within the studies that the people in comparator groups were allowed to take their usual acute headache medicines such as triptans. In addition to these limitations, the shortcomings of the included studies and underlying evidence base as mentioned in the individual studies were further limitations. Potential publication bias may also be a problem. It is possible that any study that did not find positive evidence of the use of other chronic migraine treatments did not conduct an economic evaluation or, if they did, this may not have been published.

## Conclusion

Evidence to support the cost-effectiveness of pharmacological treatments of chronic migraine in the adult population using Botox and CGRP inhibitors such as Erenumab, Fremanezumab and Galcanezumab, were identified. Based on the findings from the review, Botox and CGRPs, both were cost-effective compared to placebo; although the CGRPs had more incremental economic benefits compared to Botox, the ICERs were above the most common willingness-to-pay thresholds. CGRPs might provide an acceptable cost-effective prophylactic treatment for chronic migraine including for participants for whom the other treatments including Botox have been unsuccessful. Further research is needed to better characterise the data to adequately structure and parameterise an economic model to support decision-making for chronic migraine therapies.

## Supplementary Information

**Additional file 1: Appendix 1.** MEDLINE Search Strategy.

## Data Availability

The datasets used and/or analysed during the current study are available from the corresponding author on reasonable request.

## Abbreviations

CM: Chronic migraine
EM: Episodic migraine
CGRP: Calcitonin Gene-Related Peptide
EQ-5D: European-Quality of Life Five dimensions
HRQoL: Health-Related Quality of Life
GDP: Gross domestic product
IBMS: International Burden of Migraine Study
ICER: Incremental cost-effectiveness ratio
HIT-6: Headache Impact Test
MIDAS: Migraine Disability Assessment
MSQ: Migraine specific questionnaire
MMD: Monthly migraine days
NOK: Norwegian Krone
PREEMPT: Patients in The Phase III REsearch Evaluating Migraine Prophylaxis Therapy
PSA: Probabilistic sensitivity analyses
QALY: Quality-adjusted life year
SEK: Swedish Krona
SC: Supportive care
NICE: National Intitute for Health and Care Excellence
CADTH: Canadian Agency for Drugs and Technologies in Health

## References

[1] Steiner T, Stovner L, Jensen R, eta al (2020) Migraine remains second among the world’s causes of disability, and first among young women: findings from GBD2019. J Headache Pain 21:137. 10.1186/s10194-020-01208-0 10.1186/s10194-020-01208-0 PMC7708887 33267788

[2] Stovner LJ, Nichols E, Steiner TJ, Abd-Allah F, Abdelalim A, Al-Raddadi RM et al (2018) Global, regional, and national burden of migraine and tension-type headache, 1990–2016: a systematic analysis for the global burden of disease study 2016. Lancet Neurol 17(11):954–976 10.1016/S1474-4422(18)30322-3 PMC6191530 30353868

[3] Steel N, Ford JA, Newton JN, Davis AC, Vos T, Naghavi M (2018). Changes in health in the countries of the UK and 150 English local authority areas 1990–2016: a systematic analysis for the global burden of disease study 2016. Lancet.

[4] International Headache Society (2018). Headache classification Committee of the International Headache Society (IHS) the international classification of headache disorders, asbtracts. Cephalalgia..

[5] Berg J (2004). Economic Evidence in migraine and other headaches: a review. Eur J Health Econ.

[6] Lanteri-Minet M (2014). Economic burden and costs of chronic migraine. Curr Pain Headache Rep.

[7] Lantéri-Minet M, Duru G, Mudge M, Cottrell S (2011). Quality of life impairment, disability and economic burden associated with chronic daily headache, focusing on chronic migraine with or without medication overuse: a systematic review. Cephalalgia..

[8] Yu J, Goodman MJ, Oderda GM (2009). Economic evaluation of pharmacotherapy of migraine pain: a review of the literature. J Pain Palliat Care Pharmacother.

[9] Adelman JU, Adelman LC, Von Seggern R (2002). Cost-effectiveness of antiepileptic drugs in migraine prophylaxis. Headache..

[10] Brown J, Papadopoulos G, Neumann P, Price M, Friedman M, Menzin J (2006). Cost-effectiveness of migraine prevention: the case of topiramate in the UK. Cephalalgia..

[11] Brown JS, Papadopoulos G, Neumann PJ, Friedman M, Miller JD, Menzin J (2005). Cost-effectiveness of topiramate in migraine prevention: results from a pharmacoeconomic model of topiramate treatment. Headache.

[12] Mahon R, Huels J, Hacking V, Cooney P, Danyliv A, Vudumula U (2020). Economic evaluations in migraine: systematic literature review and a novel approach. J Med Econ.

[13] Moher D, Liberati A, Tetzlaff J, Altman DG, Group* P (2009). Preferred reporting items for systematic reviews and meta-analyses: the PRISMA statement. Ann Intern Med.

[14] Husereau D, Drummond M, Augustovski F, de Bekker-Grob E, Briggs AH, Carswell C et al (2022) Consolidated Health Economic Evaluation Reporting Standards 2022 (CHEERS 2022) statement: updated reporting guidance for health economic evaluations. Int J Technol Assess Health Care 38:e13, 1–7 10.1017/S0266462321001732 35007499

[15] Philips Z, Ginnelly L, Sculpher M, Claxton K, Golder S, Riemsma R (2004). Review of guidelines for good practice in decision-analytic modelling in health technology assessment. Health technology assessment (Winchester, England).

[16] Batty AJ, Hansen RN, Bloudek LM, Varon SF, Hayward EJ, Pennington BW (2013). The cost-effectiveness of onabotulinumtoxinA for the prophylaxis of headache in adults with chronic migraine in the UK. J Med Econ.

[17] Giannouchos TV, Mitsikostas DD, Ohsfeldt RL, Vozikis A, Koufopoulou P (2019). Cost-effectiveness analysis of Erenumab versus OnabotulinumtoxinA for patients with chronic migraine attacks in Greece. Clin Drug Invest.

[18] Hansson-Hedblom A, Axelsson I, Jacobson L, Tedroff J, Borgstrom F (2020). Economic consequences of migraine in Sweden and implications for the cost-effectiveness of onabotulinumtoxinA (Botox) for chronic migraine in Sweden and Norway. J Headache Pain.

[19] Hollier-Hann G, Curry A, Onishchenko K, Akehurst R, Ahmed F, Davies B (2020). Updated cost-effectiveness analysis of onabotulinumtoxinA for the prevention of headache in adults with chronic migraine who have previously received three or more preventive treatments in the UK. J Med Econ.

[20] Lipton RB, Brennan A, Palmer S, Hatswell AJ, Porter JK, Sapra S (2018). Estimating the clinical effectiveness and value-based price range of erenumab for the prevention of migraine in patients with prior treatment failures: a US societal perspective. J Med Econ.

[21] Mahon R, Lang A, Vo P, Huels J, Cooney P, Danyliv A (2021). Cost-effectiveness of Erenumab for the preventive treatment of migraine in patients with prior treatment failures in Sweden. Pharmacoeconomics..

[22] Ruggeri M, Carletto A, Marchetti M (2013). Cost-effectiveness of onabotulinumtoxinA for the prophylaxis of chronic migraine. [Italian, English]. PharmacoEconomics - Italian Research Articles.

[23] Sussman M, Benner J, Neumann P, Menzin J (2018). Cost-effectiveness analysis of erenumab for the preventive treatment of episodic and chronic migraine: results from the US societal and payer perspectives. Cephalalgia..

[24] Vekov T, Izmaylov A (2019). Cost-effectiveness analysis of CGRP inhibitors for treatment of patients with chronic or episodic migraine. [Bulgarian]. Gen Med.

[25] Canadian Agency for Drugs and Technologies in Health (2019) CADTH Common Drug Review: Pharmacoeconomic Review Report for OnabotulinumtoxinA (Botox). CADTH, Ottawa

[26] Canadian Agency for Drugs and Technologies in Health (2019) CADTH Common Drug Review: Pharmacoeconomic Review Report for Erenumab (Aimovig). CADTH, Ottawa

[27] Institute for Clinical and Economic Review (2018) Calcitonin gene-related peptide (CGRP) inhibitors as preventive treatments for patients with episodic or chronic migraine: effectiveness and value - final Evidence report. ICER, Boston

[28] National Institute for Health and Care Excellence (2019) Single technology appraisal: Fremanezumab for preventing migraine [ID1368] - committee papers. NICE, London 40455896

[29] National Institute for Health and Care Excellence (2019) Single technology appraisal: Erenumab for preventing migraine [ID1188] - committee papers. NICE, London 40737473

[30] National Institute for Health and Care Excellence (2020) Single technology appraisal: Galcanezumab for preventing migraine [ID1372] - committee papers. NICE, London 40875874

[31] Warwick Evidence (2011) Botulinum toxin type a for the prophylaxis of headaches in adults with chronic migraine: a single technology assessment. NICE, Coventry

[32] Doane MJ, Gupta S, Vo P, Laflamme AK, Fang J (2019). Associations between headache-free days and patient-reported outcomes among migraine patients: a cross-sectional analysis of survey data in Europe. Pain Ther.

[33] Martelletti P, Schwedt TJ, Lanteri-Minet M, Quintana R, Carboni V, Diener H-C (2018). My migraine voice survey: a global study of disease burden among individuals with migraine for whom preventive treatments have failed. J Headache Pain.

