# Supplementary material for: A systematic review of economic evaluations of pharmacological treatments for adults with chronic migraine
Source: J Headache Pain. 2022 Sep 16;23(1):122. doi: 10.1186/s10194-022-01492-y (PMC9479409; doi:10.1186/s10194-022-01492-y)
Supplement: Supplementary file 1 — Additional file 1: Appendix 1. MEDLINE Search Strategy. [file 10194_2022_1492_MOESM1_ESM.docx]

**Appendix 1: MEDLINE Search Strategy**

Search strategies for other databases/sources are available on request from the authors.

**MEDLINE (via Ovid)**

Date searched: 06/09/21
Database: Ovid MEDLINE(R) ALL <1946 to September 03, 2021>

Search Strategy:

--------------------------------------------------------------------------------

1 (headache* or head ache* or migrain* or cephalgi* or cephalalgi* or hemicrani*).ab,kf,ti. (112847)

2 Headache/ or exp Headache Disorders/ (61218)

3 1 or 2 [population: migraine/headache] (124069)

4 (((calcitonin gene-related peptide or CGRP) adj5 (antibod* or antagon* or inhibit* or block*)) or anti-CGRP or anti-calcitonin gene-related peptide or monoclonal antibod* or mAb or mAbs or moAb or moAbs).ab,kf,ti. (216382)

5 Calcitonin Gene-Related Peptide/ai (436)

6 Antibodies, Monoclonal/ or Antibodies, Monoclonal, Humanized/ (216971)

7 Calcitonin Gene-Related Peptide Receptor Antagonists/ (700)

8 (erenumab or galcanezumab or fremanezumab or eptinezumab).ab,kf,ti,nm. (506)

9 (rimegepant or ubrogepant or atogepant or gepant?).ab,kf,ti,nm. (213)

10 exp Botulinum Toxins/ (17099)

11 (botulin* adj toxin*).ab,kf,ti,nm. (21932)

12 (botulinum* or botox* or onabotulinum*).ab,kf,ti,nm. (25143)

13 (antidepress* or anti depress*).ab,kf,ti. (73848)

14 exp Antidepressive Agents/ (153091)

15 (amitriptyline or venlafaxine or mirtazapine or duloxetine).ab,kf,ti,nm. (17952)

16 exp "Serotonin and Noradrenaline Reuptake Inhibitors"/ (5001)

17 (SNRI or SNRIs or (serotonin adj2 (noradrenaline or norepinephrine) adj reuptake inhib*)).ab,kf,ti. (2907)

18 exp Angiotensin-Converting Enzyme Inhibitors/ (45311)

19 (Angiotensin Converting Enzyme Inhibit* or ACE inhibit*).ab,kf,ti. (37925)

20 acei.ab,kf,ti. (4337)

21 lisinopril.ab,kf,ti,nm. (3085)

22 ((angiotensin receptor or angiotensin II receptor) adj (block* or antagon*)).ab,kf,ti. (14463)

23 (ARB or ARBs).ab,kf,ti. (7863)

24 exp Angiotensin Receptor Antagonists/ (25388)

25 candesartan.ab,kf,ti,nm. (3374)

26 ((beta adj3 block*) or betablock*).ab,kf,ti. (55677)

27 ((adrenergic or adrenoreceptor* or adrenoceptor*) adj3 (antagonist* or block*)).ab,kf,ti. (34501)

28 exp Adrenergic beta-Antagonists/ (85429)

29 (propranolol or metoprolol or timolol or atenolol or nadolol or nebivolol or pindolol).ab,kf,ti,nm. (67109)

30 (calcium adj2 (block* or antagonis* or inhibit*)).ab,kf,ti. (41544)

31 (CCB or CCBs).ab,kf,ti. (2617)

32 exp Calcium Channel Blockers/ (88521)

33 (flunarizine or verapamil).ab,kf,ti,nm. (27699)

34 (anticonvuls* or antiepilep* or anti convuls* or anti epilep*).ab,kf,ti. (53578)

35 exp Anticonvulsants/ (147133)

36 (topiramate or valproate or divalproex or valproic acid or gabapentin).ab,kf,ti,nm. (31187)

37 Pizotyline/ (250)

38 (pizotifen or pizotyline).ab,kf,ti,nm. (418)

39 (alpha adj4 agonist*).ab,kf,ti. (15366)

40 exp Adrenergic alpha-Agonists/ (164048)

41 (clonidine or guanfacine).ab,kf,ti,nm. (19179)

42 4 or 5 or 6 or 7 or 8 or 9 or 10 or 11 or 12 or 13 or 14 or 15 or 16 or 17 or 18 or 19 or 20 or 21 or 22 or 23 or 24 or 25 or 26 or 27 or 28 or 29 or 30 or 31 or 32 or 33 or 34 or 35 or 36 or 37 or 38 or 39 or 40 or 41 [Interventions: named drugs/drug classes or types] (1098078)

43 Economics/ (27362)

44 exp "Costs and Cost Analysis"/ (248833)

45 Economics, Nursing/ (4006)

46 Economics, Medical/ (9151)

47 Economics, Pharmaceutical/ (3015)

48 exp Economics, Hospital/ (25285)

49 Economics, Dental/ (1919)

50 exp "Fees and Charges"/ (30859)

51 exp Budgets/ (13884)

52 budget*.ti,ab,kf. (32036)

53 (economic* or cost or costs or costly or costing or price or prices or pricing or pharmacoeconomic* or pharmaco-economic* or expenditure or expenditures or expense or expenses or financial or finance or finances or financed).ti,kf. (248167)

54 (economic* or cost or costs or costly or costing or price or prices or pricing or pharmacoeconomic* or pharmaco-economic* or expenditure or expenditures or expense or expenses or financial or finance or finances or financed).ab. /freq=2 (323416)

55 (cost* adj2 (effective* or utilit* or benefit* or minimi* or analy* or outcome or outcomes)).ab,kf. (179847)

56 (value adj2 (money or monetary)).ti,ab,kf. (2646)

57 exp models, economic/ (15779)

58 economic model*.ab,kf. (3649)

59 markov chains/ (15222)

60 markov.ti,ab,kf. (24937)

61 monte carlo method/ (30091)

62 monte carlo.ti,ab,kf. (53356)

63 exp Decision Theory/ (12574)

64 (decision* adj2 (tree* or analy* or model*)).ti,ab,kf. (28316)

65 43 or 44 or 45 or 46 or 47 or 48 or 49 or 50 or 51 or 52 or 53 or 54 or 55 or 56 or 57 or 58 or 59 or 60 or 61 or 62 or 63 or 64 [economic evaluations/cost/economic models filter] (791472)

66 3 and 42 and 65 [population + named drug interventions + economic filter] (209)

67 exp Migraine Disorders/dt, pc (9891)

68 "migrain*".ab,hw,kf,ti. (42481)

69 ((prevent* or prophyla*) adj2 (treatment? or therap* or medication? or drug?)).ab,hw,kf,ti. (173556)

70 ((pharmacolog* or pharmaceutical or drug? or medical) adj1 (treatment? or therap* or management)).ab,hw,kf,ti. (455613)

71 68 and (69 or 70) (4510)

72 67 or 71 (12167)

73 65 and 72 [economics filter + general terms for migraine prevention/drug treatment] (477)

74 66 or 73 (568)

The migraine/headache search terms (lines 1-3) and botox search terms (lines 10-12) are based on those used in:

Herd  CP, Tomlinson  CL, Rick  C, Scotton  WJ, Edwards  J, Ives  N, Clarke  CE, Sinclair  A. Botulinum toxins for the prevention of migraine in adults. Cochrane Database of Systematic Reviews 2018, Issue 6. Art. No.: CD011616. DOI: 10.1002/14651858.CD011616.pub2.

The search filter for economic and cost studies (lines 43-65) is the CADTH filter for Economic Evaluations/Cost/Economic Models – OVID Medline:

Strings attached: CADTH database search filters [Internet]. Ottawa: CADTH; 2016. Available from: www.cadth.ca/resources/finding-evidence/
